# Biostatistics: a fundamental discipline at the core of modern health data science

**DOI:** 10.5694/mja2.50372

**Published:** 2019-10-27

**Authors:** Katherine J Lee, Margarita Moreno‐Betancur, Jessica Kasza, Ian C Marschner, Adrian G Barnett, John B Carlin

**Affiliations:** ^1^ Murdoch Children's Research Institute Melbourne VIC; ^2^ University of Melbourne Melbourne VIC; ^3^ Monash University Melbourne VIC; ^4^ NHMRC Clinical Trials Centre University of Sydney Sydney NSW; ^5^ Institute of Health and Biomedical Innovation Queensland University of Technology Brisbane QLD

**Keywords:** Biostatistics, Epidemiology

The value of our health and medical research investment is at risk unless we foster the discipline of biostatistics

Every year, Australia's National Health and Medical Research Council (NHMRC) spends around $800 million on medical and public health research,^1^ much of which depends critically on the correct analysis and interpretation of data. We argue here that the value of our health research investment, in terms of improved health and lives saved, is at risk unless serious attention is paid to fostering the core scientific discipline of biostatistics. This risk is heightened by the expansion of research possibilities offered by the era of big data, which is rapidly enhancing the availability and scale of new information, necessitating ever deeper understanding of statistical issues and computational tools. Concerns surrounding the inadequate foundations of biostatistics in Australia were raised in a statement emanating from the International Society for Clinical Biostatistics conference held in Melbourne in August 2018 (in conjunction with the Australian Statistical Conference), the largest gathering of research biostatisticians that has ever occurred in Australia.^2^

## The problem

Statistical reasoning provides the theoretical basis for extracting knowledge from data in the presence of variability and uncertainty. It is a critical element of most empirical research in public health and clinical medicine, with the best studies incorporating biostatistical input on aspects from study design to data analysis and reporting. Biostatistical methods underpin key public health research disciplines, such as epidemiology and health services research, a role that reflects the core nature of the discipline of biostatistics. Similarly, bioinformatics and computational biology are important new areas in data‐intensive biomedical research that are underpinned by statistical concepts and methods, along with components heavily informed by other core disciplines such as computer science and mathematics. The critical role of biostatistics was affirmed in a recent review of the scale of waste and inefficiency in health research, which observed that, “These issues [of poor study design, conduct and analysis] are often related to misuse of statistical methods, which is accentuated by inadequate training in methods,”^3^ echoing similar observations made over two decades earlier.^4^

Importantly, biostatistics, as a subdiscipline of statistics (arguably, the original “data science”^5^), is an established scientific discipline of its own and is not simply a toolkit of techniques that need to be used correctly. Sound biostatistical work requires not only an understanding of mathematics, probability and sources of bias, which underpin statistical theory and methods, but also (and increasingly) extensive technical skills, including computing. In‐depth training is needed to develop these skills along with the understanding required to conceptualise problems and navigate the tricky waters between real‐world health questions and complex techniques. As noted in a recent review, such training would be very difficult to achieve for most clinicians.^6^ Superficial understanding of statistics can easily lead to unscientific practice (recently characterised as “cargo‐cult statistics”^7^) and may be seen as responsible in large part for the current “crisis of reproducibility” in research.^8^ A prominent example is the evolution of beliefs concerning the risk of cardiovascular disease associated with postmenopausal oestrogen therapy. Influential observational studies in the late 1990s claimed to demonstrate evidence of reduced risk of heart attacks, a conclusion that was contradicted by a major randomised trial.^9^ Careful re‐analysis of the observational data, guided by contemporary statistical thinking about confounding and time‐dependent changes in risk, produced results that were similar to the randomised trial.^10^

The emerging era of big data heightens the need for biostatistical expertise, with more decision makers and researchers aiming to extract value from complex messy data, and increasing use of packaged software by individuals with insufficient understanding of the underlying methods. Big data require both an advanced understanding of fundamental statistical concepts and methods, including recent developments in causal reasoning,^11^ as well as enhanced capacity in computational tools such as dimensionality reduction, distributed processing, machine learning and natural language processing. More data do not necessarily mean better data, and more analytics does not necessarily mean better science, as the quality and reproducibility of research findings will remain highly dependent on the design of the data collection, an understanding of associated limitations and resulting biases, as well as appropriate analytical methods.^12^, ^13^

## Necessary steps

Successful establishment of biostatistics as a core discipline within academic health and medical research requires recognition of biostatistics as an academic discipline, central to the intellectual infrastructure of the broader research enterprise. This implies the need for structures that support a range of levels of biostatistical work, from non‐specialists such as clinicians, to masters level biostatistics graduates and doctoral students, through to postdoctoral researchers and research leaders in biostatistical methodology. The need for academic activity across this range is similar in other areas of science, but is widely overlooked for biostatistics because of the tendency to regard the field as simply a toolkit of techniques rather than an evolving research discipline of its own. Biostatistical research develops and evaluates rigorous methods for drawing conclusions from new study designs and new data types, an extensive process that involves mathematical derivations and conceptualisations, simulation studies, detailed case studies, and translation of the newly developed methods for use by other researchers. As an example of the key role of new statistical methods, the development of marginal structural models was critical in the wave of research into antiretrovirals for the treatment of human immunodeficiency virus infection, by enabling the appropriate handling of time‐dependent confounding in treatment decisions based on CD4 cell count levels that are themselves affected by treatment.^14^ Experience in methodological research is also an essential component in the training of future biostatistical leaders.

As for any academic discipline, in order to support the continued development of extensive training pathways for biostatisticians, we need clearly identified departmental structures within our institutions. These should provide hubs of sufficient critical mass to enable transfer of expertise and knowledge within and between the multiple levels of activity, from non‐specialists to research leaders. These hubs need to be embedded within schools of public health, medicine and health sciences, and their partner institutes, and should be led by biostatisticians who are active in methodological research.

## The international situation and Australia's position

The fundamental importance of biostatistics to health and medical research has been recognised in other countries. In the United States, many major universities have departments of biostatistics that were established in the 1970s through funding of biostatistical research training programs by the National Institutes of Health, with a call for a renewed effort to expand biostatistical training programs in 2006.^15^ In a similar vein, the Medical Research Council in the United Kingdom has long funded a national centre in biostatistical methodology — the Medical Research Council's Biostatistics Unit — and, since 2009, a number of methodology hubs whose core research agenda is statistical methodology (http://www.methodologyhubs.mrc.ac.uk). There are also dedicated streams of funding for methodological research. In continental Europe, the Integrated Design and Analysis of small population group trials (IDeAl) consortium received €3 million over 2013–2019 from the European Union's Framework for Research and Innovation funding program to develop new design and analysis methodologies.^16^ Long term investment in biostatistical research in these nations means that they are much better placed in terms of methodological infrastructure underpinning their medical research. For example, modern trialists are moving towards adaptive trials and, in particular, platform trials, yet researchers developing such trials in Australia are reliant on biostatistical expertise from overseas.

In contrast to Europe and the US, there has never been systematic investment in the development of biostatistics in Australia, either in universities or via national funding schemes. None of the major universities has a department of biostatistics; instead, there are many small groups (or even just individuals), often only loosely connected with each other or within departments or schools that are dominated by disciplines other than medicine and public health. For example, all of the Group of Eight universities have structures that link statistics with mathematics or business, which inhibits the linkage between biostatistical and medical research that is critical for achieving excellence in the planning, conduct and analyses of medical research studies. This landscape is just beginning to change at the University of Melbourne and Monash University, with recent initiatives for the recruitment of research biostatisticians at a range of levels. Among the medical research institutes, the Clinical Epidemiology and Biostatistics Unit at Murdoch Children's Research Institute provides an example of a successful biostatistics core, with academic leadership underpinned by a methodological research program and a “hub and spokes” model whereby staff hold joint positions with our group and the research groups they support.

With regards to funding, we are aware of only one example in Australia of direct funding of a group of biostatisticians with a critical mass and a research base in biostatistics: the Victorian Centre for Biostatistics (ViCBiostat), which was established in 2012 under an NHMRC Centre of Research Excellence grant. However, funding of this centre ceased in 2017. The only other possible avenue for funding of biostatistical research in the current climate is short term project and investigator grants, but this is not a sustainable avenue to ensure an ongoing critical mass, particularly given that the downstream impact of methodological research will always tend to make it less competitive than substantively focused medical research. An ongoing commitment in the form of dedicated investment in methodological research is a key requirement for developing and maintaining an essential biostatistics infrastructure.

## Potential solutions

There is unfortunately no quick solution to the problems outlined, but we suggest some steps that we believe are needed to strengthen and develop the biostatistics discipline in Australia:

- universities and research institutes need to foster the development of organisational structures with a critical mass of academic biostatisticians working both in methodology and collaborating with health researchers, as well as training opportunities and career development for biostatisticians;
- biostatistical teaching and advanced training must keep pace with the dramatic changes in the data science landscape,^11^, ^15^ to ensure that graduates have the necessary breadth of skills to support medical research in the modern era — this requires leadership from the field; for example, via the Biostatistics Collaboration of Australia (http://www.bca.edu.au); and
- funding bodies need to invest in biostatistical research; for example, by the creation and support of graduate and postdoctoral methodological training programs, to ensure the discipline can provide the base of expertise that is necessary to support medical research at internationally competitive levels.

Without investment in biostatistics at these multiple levels, the entire Australian medical research enterprise is at considerable risk of “drowning in data but starving for knowledge”.^17^

## Competing interests

No relevant disclosures.

## Provenance

Not commissioned; externally peer reviewed.
